# Artificial coiled coil biomineralisation protein for the synthesis of magnetic nanoparticles

**DOI:** 10.1038/s41467-019-10578-2

**Published:** 2019-06-28

**Authors:** Andrea E. Rawlings, Lori A. Somner, Michaela Fitzpatrick-Milton, Thomas P. Roebuck, Christopher Gwyn, Panah Liravi, Victoria Seville, Thomas J. Neal, Oleksandr O. Mykhaylyk, Stephen A. Baldwin, Sarah S. Staniland

**Affiliations:** 1 Department of Chemistry, University of Sheffield, Sheffield, S3 7HF UK; 2 School of Physics and Astronomy, University of Leeds, Leeds, LS2 9JT UK; 3 Faculty of Biological Sciences, University of Leeds, Leeds, LS2 9JT UK

**Keywords:** Protein design, Biomaterials - proteins, Protein aggregation

## Abstract

Green synthesis of precise inorganic nanomaterials is a major challenge. Magnetotactic bacteria biomineralise magnetite nanoparticles (MNPs) within membrane vesicles (magnetosomes), which are embedded with dedicated proteins that control nanocrystal formation. Some such proteins are used in vitro to control MNP formation in green synthesis; however, these membrane proteins self-aggregate, making their production and use in vitro challenging and difficult to scale. Here, we provide an alternative solution by displaying active loops from biomineralisation proteins Mms13 and MmsF on stem-loop coiled-coil scaffold proteins (Mms13cc/MmsFcc). These artificial biomineralisation proteins form soluble, stable alpha-helical hairpin monomers, and MmsFcc successfully controls the formation of MNP when added to magnetite synthesis, regulating synthesis comparably to native MmsF. This study demonstrates how displaying active loops from membrane proteins on coiled-coil scaffolds removes membrane protein solubility issues, while retains activity, enabling a generic approach to readily-expressible, versatile, artificial membrane proteins for more accessible study and exploitation.

## Abstract (web summary)

Proteins have been used in the synthesis of magnetic nanoparticles but issues with aggregation limit this application. Here, the authors report on the synthesis of coiled proteins that display the active loop of the natural proteins to avoid aggregation and investigate the application in nanoparticle synthesis.

## Introduction

The pursuit of designer magnetic nanoparticles has spanned several decades and has lately warranted increasing research activity as the number of technological applications for nanomaterials grows^1,2^. Applications range from high density recording media for information storage^3^, ferrofluids for high frequency electronics^4^, as well as several biomedical^5–7^ such as MRI contrast enhancing agents^8,9^, site-specific chemo^10^, and hyperthermic^11^ cancer treatments, as well as targeted drug delivery systems^9,12^. All nanomagnetic applications have specific requirements for the nanoparticles they utilise with respect to quality, size, shape, coatings, stability, and composition^2,11,13^. The ultimate goal within this research area is to synthesise magnetic nanoparticles with precise nanoscale control over these properties. While this may be possible for a range of sizes and some morphologies, the chemical methods generally utilise toxic precursors and solvents, as well as harsh reaction conditions, which raises issues with biocompatibility of the resulting materials and the environmental sustainability of the process. Some researchers are developing alternative strategies, for example by fine tuning reaction conditions in room temperature conditions^14^. However, ambient, biologically compatible conditions typically produce ill-defined particles over a large size range^2^. Furthermore, there are many desirable shapes and sizes which are extremely difficult to access synthetically^15^.

**Fig. 1:**
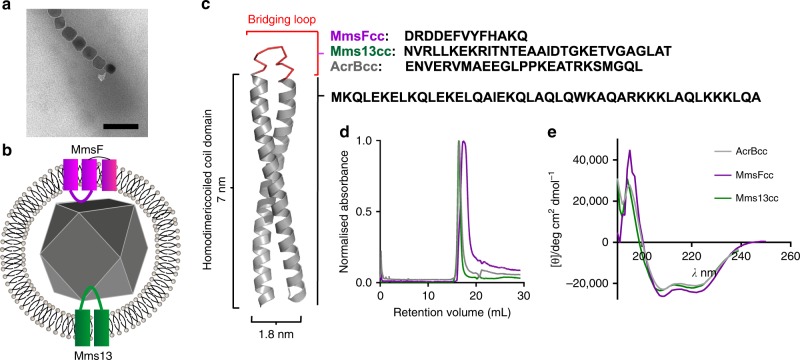
Design and characterisation of the coiled coil proteins. **a** TEM image of magnetosomes inside an *M. magneticum* AMB-1 cell (scale bar is 100 nm) and **b** a schematic of the magnetosome membrane and magnetite crystal showing the topology of Mms13 (green) and MmsF (purple) in the membrane with the transmembrane region shown as cylinders. **c** Robetta model^60^ of the coiled coil construct generated in PyMOL^68^, grey is the homodimeric coiled coil and red is the variable bridging loop comprising sequences from MmsF, Mms13, and AcrB; **d** size exclusion chromatography and **e** circular dichroism spectra of MmsFcc, Mms13cc, and AcrBcc

Achieving reproducible control on such small length scales using environmentally sustainable chemistry has presented a formidable challenge for researchers, leading us to look towards biology for a solution. Living organisms form intricate inorganic materials with nanoscale precision through biomineralisation. Nature biomineralises these materials from the bottom up; producing unparalleled control over the materials size, shape, and composition^1^. One such material is the magnetic nanoparticles of magnetite, produced in magnetotactic bacteria (MTB)^16^. These bacteria contain unique organelles, termed magnetosomes (Fig. 1a)^17–19^. The magnetosome, an intracellular vesicle structure, is made of lipid membrane and harbours an array of different proteins^20–22^. These unique proteins control and mediate key aspects of the particle formation; from facilitating iron influx across the membrane to nucleation of iron ions, as well as controlling the final morphology of the mature nanoparticle^18^. This latter group of proteins, able to influence the final morphology, size, and characteristics of the mature crystal, represent a promising source of control agent additives to regulate the formation of synthetic magnetite nanoparticles (MNPs). This has been demonstrated with several such magnetosome membrane specific (Mms) proteins, mediating the crystallisation of highly homogeneous MNPs when added to a simple, green, magnetite chemical precipitation reaction^23–25^. Mms6 was found tightly bound to the magnetite crystal within the magnetosome and so identified as a good candidate to control a reaction in vitro^23^. Mms6 was the first protein to be used in synthetic magnetite formation reactions^23,26–28^, and was found to increase the particle’s crystallinity, as well as constraining the size to 21–22 nm in most cases^28^. Mms13 (MamC) was also found tightly bound to the mineral in vivo^23^, and this too has been purified and added to magnetite precipitation, resulting in an increase in particle size^25^. Mms6 from *Magnetospirillum magneticum* AMB-1, and the Mms13 homologue, MamC, in *Magnetococcus marinus*, have been shown to bind iron ions, indicating a possible role in magnetite nucleation^23,29–32^. Genetic studies show that MmsF could be another protein of interest. The *mmsF* gene is found in the same gene cluster as *mms6*, and knock out mutations of *mmsF* and the whole gene cluster in *M. magneticum* AMB-1 resulted in smaller misshapen magnetosomes^33^. However, reintroducing *mmsF* into the strain with the whole gene cluster missing, rescued the formation of the wild-type magnetosomes, showing MmsF to be independently crucial in controlling morphology^33^. We found that MmsF could also affect the crystallisation of magnetite MNPs when added to a simple room temperature co-precipitation synthesis, tightly defining the morphology^24^.

However, a major bottleneck to exploitation is the transmembrane spanning nature of these proteins which have one, two, and three such regions in Mms6, Mms13, and MmsF, respectively^34^. Membrane proteins are extremely hydrophobic, and therefore challenging to both purify and utilise in aqueous solution without the addition of stabilising detergents or other amphiphilic additives^35^. Some of the magnetosome proteins, although possessing transmembrane helical regions, are in fact able to be refolded from inclusion bodies when over-expressed in *Escherichia coli* to form water-soluble assemblies (Mms6 and MamC)^23,25^, or can even be expressed as water-soluble multi-meric structures directly (MmsF)^24^. However, the yield of these proteins is often low, and the high degree of self-assembly makes structural and functional studies more challenging.

The Mms proteins, which interact with the magnetite crystal directly, do so via the parts of the polypeptide chain which reside within the magnetosome lumen. In the case of MmsF and Mms13, this sequence most likely lies within the intra-protein loop connecting transmembrane helices 1 and 2^34,36^. These short peptide sequences would normally be presented to the magnetite nanocrystal as a constrained loop, anchored in place by two transmembrane spanning helices (Fig. 1b). Both of these proteins have been shown to be active in synthetic magnetite precipitation reactions in their full length form^24,25^, where they improve the uniformity of the nanoparticle product. However, the production and purification of such proteins is low yielding and not trivial. There is a compelling need to develop more easily expressible, soluble, high yielding, robust proteins, with the same or similar functionality, to act as viable additives for the green synthesis of MNP. There has been progress towards this using many different approaches such as combinatorial and screening methodologies^37,38^.

Scaffold proteins have generated significant interest over recent years as researchers seek to engineer proteins with unique properties. Most scaffold proteins feature a highly stable core structure, able to tolerate significant modification of surface exposed loops. Well characterised examples include DARPins^39^, Affibodies^40^, and Adhirons^41^. Recently, Adhirons and Maltose binding protein (MBP) have been used successfully to display both artificial and naturally occurring magnetite interacting peptides^42–44^. Coiled coils represent a structural protein motif that is widespread in nature^45,46^. They comprise two or more alpha-helices which wrap around one another in a supercoiled assembly^47^. The helices can adopt either a parallel or antiparallel arrangement depending on the N to C terminal direction of each helix with respect to the other. The driving force of the assembly process in aqueous solution is the burying of the hydrophobic residues arranged along a single interior face of each helix. This is achieved by programming the coiled coil assembly into the primary structure of the peptide via a heptad repeat sequence; placing hydrophobic, charged, and polar residues at strategic sites along its length^48^. Designed coiled coils have been used for a number of innovative applications including as MRI contrast reagents and molecular motors^49,50^. Many examples of engineered coiled coils are available, where the designers have selected parallel or antiparallel coils, and dimeric or multimeric intermolecular and intramolecular assemblies, depending on the desired structure and application^48,51–54^. An antiparallel arrangement of helices lends itself to the formation of a hairpin, helix-turn-helix structure, if both helices are part of the same molecule and their termini are bridged by a peptide (Fig. 1c)^55^.

Here, we report a simple, artificial biomineralisation protein, based on the active sequences from multi-transmembrane Mms protein loops. We have developed an artificial scaffold protein to display these peptide loops in an attempt to mimic the topology of the full length proteins, and exploit just those parts which are capable of interacting with magnetite directly. Our scaffold is a de novo protein architecture based upon an antiparallel coiled coil hairpin. In this study, we demonstrate the success of using this constrained bridging peptide to display the magnetite interacting loops of Mms13 and MmsF, and show that the MmsF displaying coiled coil (MmsFcc) controls particle formation in vitro comparably to MmsF, while the results with Mms13cc may allude to Mms13’s function in vivo. This stem-loop coiled coil (SLCC) scaffold could in principle be applied to other transmembrane proteins with solvent exposed loop regions, simply by modifying the linking peptide sequence. Therefore a full range of different functionalities could in theory be conferred onto this versatile scaffold, with particular significance for intractable membrane proteins.

## Results

### Production and characterisation of the coiled coil proteins

To design our SLCC scaffold we utilised a de novo peptide sequence which has previously been demonstrated to form intermolecular antiparallel coiled coil assemblies^45^. This sequence can dimerise to form a stable coiled coil structure and features a gradient of negative to positively charged residues (N to C) along the length of each coil. The presence of these charges helps to maintain, through favourable charge–charge interactions, the antiparallel, rather than parallel topology^56^. We used the TMpred server to estimate the location of membrane spanning helices in MmsF, and Mms13 from *M. magneticum* AMB-1, as well as the acriflavin efflux protein AcrB from *E. coli* as a negative control (Supplementary Fig. 1)^57^. The experimentally determined topology of Mms13^36^, and the predicted topology of MmsF^34^, allowed us to identify the loop sequences which are expected to reside within the lumen of the magnetosome and connect two transmembrane helices. The sequences of these loops were incorporated into the construct design (Fig. 1c). Models of the coiled coil construct suggest the coils are separated at the bridging loop position by 9.8 Å. This compares favourably with the 10.6 Å separation between helices 4 and 5 in AcrB, and 9.5 Å and 10.6 Å separation in the models of MmsF and Mms13, respectively, meaning the loops are likely to be displayed in a similar fashion to that expected in the native proteins. The constructs were synthesised as synthetic genes, encoding two identical coil sequences connected via the identified loop sequence. The synthesis products were transferred into *E. coli* expression vectors to produce fusions with an N-terminal polyhistidine (6xHis) tag. Overexpression of the SLCC from lactose inducible promoters in *E. coli* resulted in the production of insoluble target protein, most probably a multimeric complex of intertwining coils. To circumvent this we adopted a denaturing and on-resin refolding methodology with Ni-NTA resin. The target proteins eluted as a soluble species when imidazole was applied and in high purity, confirmed by mass spectrometry (Supplementary Fig. 2 and Supplementary Table 1). Typical yields are in the range 15–20 mg L^−1^ culture.

Circular dichroism (CD) measurements have shown that all three studied proteins consistently produced intense peaks at 220 and 208 nm which are characteristic of an α-helical structure (Fig. 1e). This suggests that the bridging loop can be readily substituted without affecting the secondary structure of the scaffold. MmsFcc demonstrates a slightly higher degree of α-helical content compared to the other two proteins^58^. This is expected due to the shorter, non-helical bridging loop of MmsFcc compared with AcrBcc and Mms13cc.

We wanted to determine if the proteins were assuming monomeric coiled coil structures, and exclude the possibility of dimers, or higher order oligomers. Using a calibrated size exclusion column, the three proteins were analysed to generate approximate molecular weights. The retention volumes of each of the proteins were consistent with the desired monomeric species (Fig. 1d), with small variances between the constructs matching the differently sized bridging loops (Fig. 1c). Reassuringly, any peaks consistent with larger assemblies were completely absent, indicating that our samples were both monomeric and homogeneous.

**Fig. 2:**
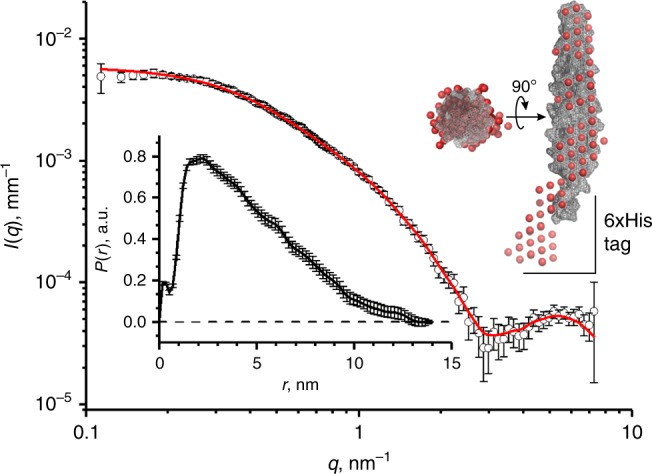
MmsFcc SAXS analysis. Experimental (open circles) and fitted (red line) SAXS patterns of MmsFcc with 6xHis tag in PBS solution (concentration 2.5 mg mL^−1^). The pair-distance distribution function, *P*(*r*), is shown as inset. A SAXS envelope of the scattering object reconstructed by ab initio shape determination (red spheres) (see details in Methods) overlapped with the protein molecule space filling Robetta model^60^ (grey mesh) both top down and side on views are shown at the top right corner of the figure^68^

However, both size exclusion chromatography and CD do not conclusively confirm the presence of rod-shaped hairpin structures. To determine both the shape and dimensions of the coiled coil constructs, small angle X-ray scattering (SAXS) measurements of purified MmsFcc with attached 6xHis tag were collected. A relatively smooth curve was fitted to the experimental SAXS data in GNOM^59^, yielding a pair-distance distribution function *P*(*r*) and a radius of gyration of the scattering objects *R*_g_ = 3.7 ± 0.2 nm (Fig. 2). The obtained *P*(*r*) pattern revealed that MmsFcc has a rod-like shape with a maximum length of about 12 nm. This is consistent with our MmsFcc structural models (Fig. 1c) generated in Robetta^60^ with the addition of a flexible 6xHis tag. Some oscillations observed in the *P*(*r*) are likely to be related to the periodic nature of the α-helical protein coil.

### Using the coiled coil proteins to mediate MNP precipitation

**Fig. 3:**
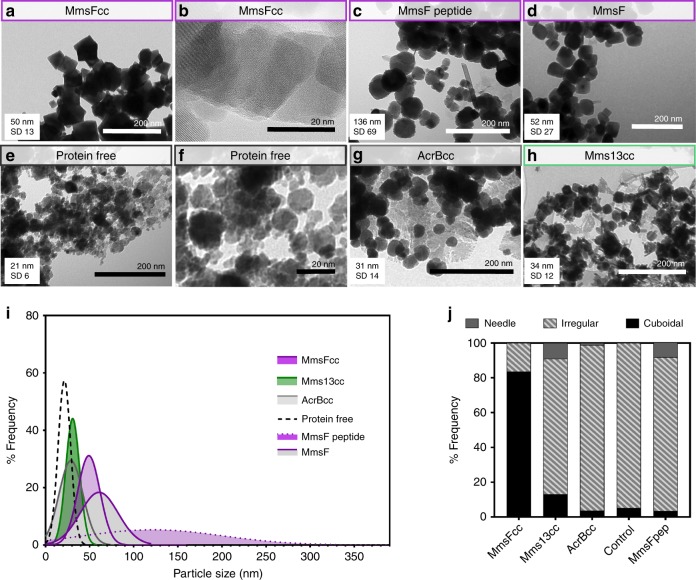
MNP analysis. Transmission electron microscopy analysis of nanoparticles formed with a variety of different protein additives. Scale bars are indicated, and the mean particle diameters and standard deviations are shown. **a** MmsFcc, **b** MmsFcc at high magnification (380,000×), **c** MmsF peptide, **d** MmsF (shown for comparison, see ref. ^24^), **e** protein free, **f** protein free at high magnification, **g** AcrBcc. **h** Mms13cc. Data = mean with standard deviation (*n* = 100) displayed in each image. **i** Histograms of particle sizes have been generated and fitted to a Gaussian distribution for clarity (see S4 for raw histograms), **j** shape analysis of particles from TEM images

**Fig. 4:**
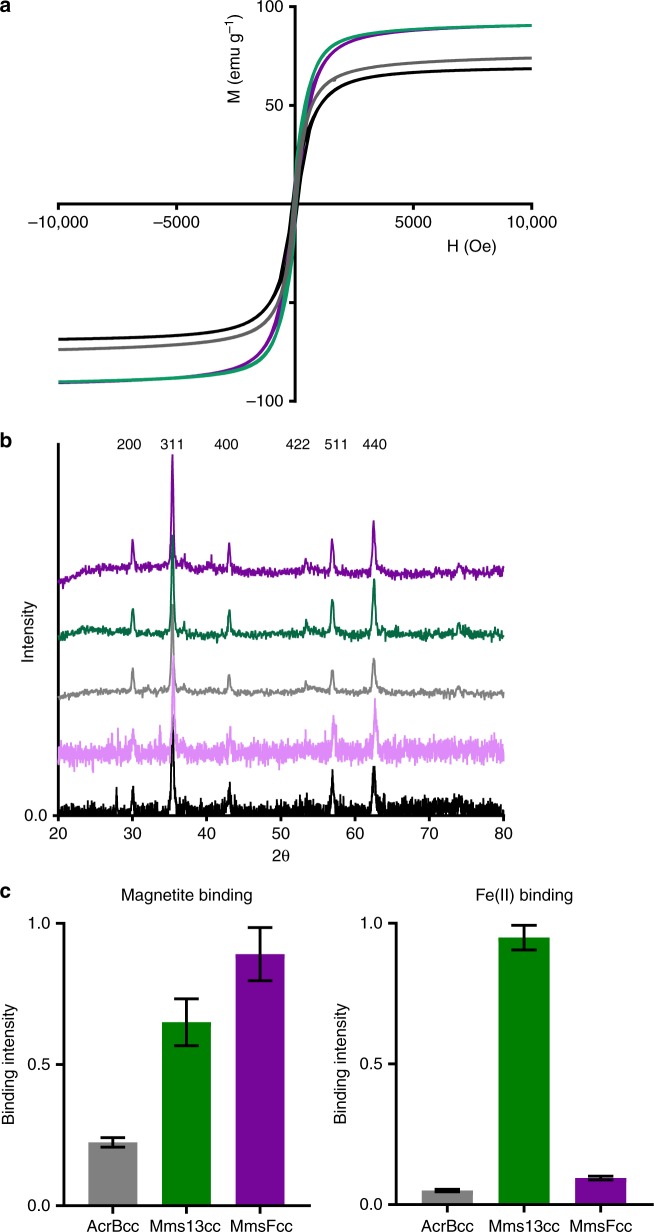
Particle analysis and protein activity. **a** Vibrating sample magnetometry (VSM) analysis of particle samples generated with the three coiled coil proteins, MmsF peptide, and a protein free control. Magnetisation (*M*) in emu g^−1^ and magnetic field strength (*H*) in Oersted are displayed. **b** Powder X-ray diffraction analysis of the same particle samples. Miller indices at the top of the XRD patterns are assigned to reflections corresponding to magnetite. The data sets are offset for clarity. **c** Magnetite nanoparticle and ferrous iron binding analysis. Data are normalised to the highest reading in each experiment. Data = mean with standard deviation (*n* = 3). In all parts, MmsFcc is depicted in purple, Mms13cc in green, AcrBcc in grey, protein free in black, and MmsF peptide in light purple

To study the effect of the coiled coil constructs on the morphology of MNPs during crystallisations, MmsFcc, Mms13cc, and the negative control protein AcrBcc, were added to magnetite co-precipitation reactions (Fig. 3a, h, g, respectively). The active flexible peptide region of MmsF was also added into co-precipitation reactions to assess the differences between an unconstrained sequence versus a constrained loop (Fig. 3c). These reactions were carried out at room temperature, using a final protein concentration of 5 µg mL^−1^. The nanoparticle products were compared to particles prepared in the absence of protein (Fig. 3e, f). Powder X-ray diffraction (XRD) indicated that all of the samples produced were most likely to be predominately magnetite (it is not possible to completely rule out the presence of the closely related iron oxide, maghemite, due to their similar crystal structure and lattice parameters). Furthermore, XRD will not resolve amorphous iron oxides or small amounts of crystalline alternative iron oxides. However, magnetometry analysis indicates MmsFcc and Mms13cc particles to be a pure magnetite product based on their saturation magnetisation, whereas AcrBcc, and the protein free sample, must comprise a greater proportion of other non-magnetic iron oxides, as their saturation magnetisation is lower (Fig. 4a, b and Table 1).

**Table 1:** Nanoparticle characterisation

MNP sample	TEM particle size (nm)^a^	Crystallite size (nm)^b^	Saturation magnetisation (emu g^−1^)
MmsFcc	50 (13)	49	90
Mms13cc	34 (12)	39	93
AcrBcc	31 (14)	35	74
MmsF peptide	136 (69)	59	54
Protein free	21 (6)	38	69

^a^Mean particle size with standard deviation provided in parentheses

^b^Calculated using the Scherrer equation and the diffraction peak from the [311] magnetite plane

Transmission electron microscopy (TEM), and subsequent particle size analysis, showed that the MmsFcc prepared particles were significantly larger (mean Ø 50 nm, standard deviation 13 nm) than either the protein free, Mms13cc, or AcrBcc particles (Fig. 3i, Supplementary Fig. 3, and Supplementary Table 2) and comparable in both size, shape, and pure composition to particles mediated by native MmsF protein (Mms13cc and AcrBcc were not statistically significantly different in size to each other). MNPs produced in the presence of MmsF^24^ are shown in Fig. 3d for comparison. The addition of MmsF unconstrained peptide resulted in a less controlled material composition (rods and plates in TEM) and population of wide size distribution (mean Ø 136 nm, standard deviation 69 nm) in stark contrast to the constrained MmsFcc. Critically, only MNP formed in the presence of MmsFcc displayed an altered apparent octahedral morphology, similar to those formed in a high-temperature synthesis or in the presence of MmsF native protein. Analysis of the lattice fringes from MmsFcc prepared particles using high-resolution TEM (Fig. 3b) confirmed the presence of (111) planes of magnetite, consistent with an octahedral morphology. This was not observed in the samples produced with any of the alternative coiled coil constructs, peptide, or from the protein free reaction.

### Probing the proteins for mechanistic insights

We wanted to understand the interaction between the proteins and the nanoparticles further, and therefore used a modified enzyme linked immunosorbent assay (ELISA) to probe binding^43^. Briefly, the purified protein was mixed with nanoparticles in a blocking buffer (casein based), and unbound protein was removed and discarded. The nanoparticles were probed with an antibody specific for polyhistidine tags, which in turn was detected using a conjugated alkaline phosphatase secondary antibody, yielding a yellow to blue colour change with BluePhos reagent. We investigated the proteins ability to bind to pre-made MNPs, as well as to precursor iron oxides which form *en route* to magnetite, namely ferric hydroxide and green rust^61^. Mms13cc and MmsFcc produced an intense, and highly intense, colour change respectively in our ELISA system for magnetite, indicating a positive binding event to this mineral (Fig. 4c and Supplementary Fig. 4). There was a subtle colour change observed with AcrBcc, suggesting a very low level interaction. This indicates that the coiled coil scaffold itself, as well as the presence of the polyhistidine tag, are not sufficient to produce a strong, positive, interaction to magnetite on their own. This allows us to localise the site of binding to the bridging loop region, effectively the parts of our constructs which are derived from the magnetotactic bacterial proteins MmsF and Mms13. MmsFcc appears to bind more strongly than Mms13cc to MNPs based on ELISA intensity. No significant interaction was observed to the precursor materials for MmsFcc and Mms13cc compared to the AcrBcc control protein (Supplementary Fig. 5).

We observed a different preference when the proteins were analysed for ferrous ion binding. In this experiment, purified proteins were spotted onto nitrocellulose membrane, before blocking with bovine serum albumin, and incubating with ferrous chloride. Metal binding was detected by the ferrous ions ability to catalyse the breakdown of hydrogen peroxide and trigger luminescence from applied luminol substrate. Binding was clearly detected with Mms13cc, with no clear response recorded for the other two coiled coil proteins tested (Fig. 4c and Supplementary Fig. 7). It therefore appears from our data that MmsFcc has a magnetite surface interaction, and Mms13cc has mainly a ferrous ion binding, and some magnetite surface interaction. This, along with the difference seen in the MNPs produced, suggests that perhaps MmsF and Mms13 have different mechanisms and roles in vivo.

## Discussion

It has been known for 15 years that some biomineralisation proteins from MTB have the remarkable capacity to direct the formation of MNPs in vitro as an additive to ‘green’ chemical synthesis^22^, with particular control over size^25,28^, shape^24,33^, and directing the composition towards pure magnetite^62^. Research interest in using active protein species to direct and tailor nanomaterial synthesis has developed further, with ever expanding ranges of materials, as well as an increased understanding of the protein interaction with the inorganic materials they form^38^. However, the major drawback to developing this biological/chemical hybrid methodology is the restrictive nature of producing, processing, and utilising membrane proteins. This is the case for magnetotactic bacterial-derived biomineralisation proteins, but is also a wider problem in the development and analysis of materials and processes involving membrane proteins. Examples are coming through in which researchers have developed peptides which are inspired by these biological processes, and have showed evidence of size and shape control in nanoparticle synthesis^38^.

In this study, we have designed a functional protein which retains the activity of the membrane protein but without the challenging membrane spanning regions. Substituting these regions for a robust, highly expressible, and soluble coiled coil scaffold, allows the active biomineralisation loop to be constrained between an antiparallel coiled coil, similar to if it were displayed on the native protein. We have shown this constraint to be essential, as the unconstrained active peptide alone has a negative impact on particle formation. We have shown that MmsFcc is able to control the morphology, size, and composition of the forming magnetite MNPs. Particles are pure magnetite and larger (average 50 nm) than control MNPs, and display octahedral crystal planes comparable to high-temperature magnetite synthesis or MmsF mediated synthesis. However, conversely to native Mms proteins, the SLCC is much more stable and readily produced to aid their use within MNP synthesis. This is evident from the higher expression levels, and as high molecular weight multi-subunit structures are not required, the SLCC can be easily and rapidly refolded using on-column methods. The SLCC also acts as a useful tool to allow the effects of the magnetite interacting loop and multi-subunit self-assembly to be decoupled.

Interestingly we did not see such a marked effect when Mms13cc was used in the magnetite co-precipitation. Mms6, Mms13, and MmsF have been studied in vitro, with Mms13 (MamC) found to have a subtle effect, by slightly increasing the MNP size^25^. However, our construct does appear to mimic this effect with particles slightly larger than those produced protein free (34 nm versus 21 nm). However, our negative control protein, AcrBcc also produces a modest increase in mean particle size (31 nm), so the effect of Mms13cc appears less significant. We cannot exclude the possibility that the scaffold is better able to match the loop structure from the shorter MmsF sequence than the much longer Mms13 sequence. Mms13 was discovered tightly bound to the magnetosome magnetite mineral at the same time as Mms6^23^. Since this discovery, it has been found that Mms6 activity relies in part on its aggregation and self-assembly to form a charged surface to bind iron ions and nucleate mineralisation^28,29,31^. The propensity to bind iron ions (present at the initiation of the reaction) rather than the growing magnetite MNP, is a hallmark of a nucleating function, as opposed to a morphological control function. From our data we see that Mms13cc can bind ferrous ions whereas MmsFcc does not, indicating a different type of control over crystallisation between these two proteins. One could extrapolate further to propose that Mms13 is a nucleation protein and as such may require the ability to self-assemble, similar to Mms6, in order to create a charged surface with multiple sites for crystal nucleation. The native Mms13 protein has the ability to aggregate, while this is missing in the Mms13cc construct, which could be the reason why no significant effect is observed in magnetite synthesis. Conversely, MmsFcc does not bind ferrous ions and has been shown to have morphological control over the MNP formation independent of protein aggregation. This comparison is further evidence for the different roles certain magnetosome proteins have in the crystallisation process and the different characteristics required to perform these roles.

The coiled coil scaffold displays the active loop region from the native protein in a similar configuration to that proposed to be displayed in the native protein. The SLCC offers a simple, soluble, scaffold to display, and topologically constrain, functional peptide loops from demanding proteins. This has far-reaching implications: firstly, it offers a platform for a more tractable way to study activity in membrane proteins, enabling in-depth characterisation and understanding. Secondly, it enables the use of previously inaccessible proteins for synthesis in biotechnology, nanotechnological, and pharmaceutical purposes.

## Methods

### Protein modelling

The coiled coil construct, MmsF, and Mms13 were modelled using Robetta^60^. The crystal structure of AcrB was obtained from the PDB (accession number: 1IWG). Structures were visualised, and measurements made, using Pymol software.

### Cloning and expression

MmsFcc, Mms13cc, and AcrBcc DNA sequences were amplified using PCR. The PCR products were subjected to restriction digest and ligated into a pPR-IBA1 expression vector. The coiled coil proteins were produced in BL21 (DE3) *E. coli* cells as fusion proteins to an N-terminal His_8_ tag. The proteins were grown in Super Broth autoinduction media, with trace elements (Formedium) for 40 h at 37 °C while shaking.

### Purification

Following autoinduction cells were harvested using centrifugation (20 min, 3,000 × *g*). The protein pellet was resuspended in phosphate buffered saline (PBS) 20% wt/vol, before using sonication to lyse the cells. The lysate was subjected to centrifugation (40 min, 12,000 × *g*) and the pellet was recovered. The pellet was resuspended in 8 M GuHCl, 50 mM Tris pH 8, and sonicated once more to fully disrupt the pellet. Following a final centrifugation step (30 min, 12,000 × *g*) the supernatant containing the soluble, denatured, protein fraction was recovered. The soluble protein was mixed with Amintra Ni-NTA resin (Expedeon, UK) and washed with decreasing concentrations of GuHCl buffer. Finally, the protein was eluted in PBS containing 300 mM Imidazole pH 7.4. The protein was dialysed against ultrapure water, and analysed by electrospray ionisation mass spectrometry.

### In vitro biomineralisation

Fe_3_O_4_ nanoparticles were synthesised using a room temperature co-precipitation reaction (RTCP) with Fe (II) and Fe (III) sulfates. The total iron concentration was 50 mM. A 0.5 M NaOH solution was introduced into the reaction vessel at a rate of 20 µL min^−1^. 5 µg mL^−1^ of protein was added at the start of the reaction. The reactions were performed with a ratio of the Fe(III) to total Fe of 0.3, and with four equivalents of sodium hydroxide added. All reactant solutions were deoxygenated before the experiment by sparging with nitrogen gas. Nitrogen was also bubbled through the reaction for its entire duration.

### Circular dichroism

The purified protein, present in ultrapure water, was diluted to a concentration of 0.18 mg mL^−1^ and insoluble species were removed via centrifugation at 12,000 × *g*. A Jasco J-810 instrument was used to collect CD spectra. The protein sample was added to a cuvette with a 2 mm path length and the wavelength scanned from 260 to 190 nm with a 1 nm slit width and at 1 s intervals at 20 °C. The process was repeated and the average (minus a protein-free baseline) was calculated.

### Small angle X-ray scattering

SAXS patterns were recorded at a synchrotron (ESRF, station ID02, Grenoble, France) following established protocols^63^. A monochromatic X-ray beam (wavelength *λ* = 0.0995 nm) and a 2D SAXS detector (Rayonix MX-170HS) were used for these experiments. A minimum camera length available at the station (0.8 m) was used to obtain data corresponding to a *q* range of 0.1–7.2 nm^−1^, where

1 $$q = \frac{{4{\mathrm{\pi }}\mathrm{sin}{\mathrm{\theta }}}}{{\mathrm{\lambda }}}$$

the modulus of the scattering vector and *θ* is half of the scattering angle. A flow-through glass capillary of 1.7 mm diameter was used as a sample holder. A maximum protein concentration of 2.5 mg mL^−1^ was used for the measurements. The data were recorded at 21 °C. In order to obtain the scattering signal of a reasonable intensity for SAXS analysis at the same time avoiding damage and aggregation of proteins caused by X-rays, a few experimental protocols with a variable exposure time, a number of frames and different pauses between the frames have been tested. It has been found that 10 frames with exposure time of 0.1 s each and 5 s pause between the frames were an optimal protocol for the SAXS data collection. SAXS measurements performed at lower *q* values (0.015 nm^−1^ < *q* < 1 nm^−1^) using a longer camera length (5 m) have also confirmed that no protein aggregation was occurring at these experimental conditions—the scattering patterns were flat at low *q* and no upturn of the scattered signal associated with a protein aggregation was observed. X-ray scattering data were reduced (integration, normalisation, and subtraction of buffer solution background scattering) using standard routines available at the beamline (software package SAXSutilities) and Irena SAS macros for Igor Pro^64^. The scattering intensity of water was used for absolute scale calibration of the SAXS data.

Nine dummy atom models were reconstructed using ab initio approach with no symmetry (space group P1) realised in DAMMIF^65^, which were then aligned and averaged in DAMAVER^66^ with no rejections and a normalised spatial discrepancy (NSD) of 1.09 ± 0.02. A visual inspection has suggested that all the models have a similar elongated shape. However, the NSD value is rather high indicating some systematic differences between the models and, perhaps, a further analysis with extra confinements associated with the protein structure would be required. Nevertheless, the most probable SAXS envelope, obtained by removing low occupancy and loosely connected atoms from the averaged model using DAMFILT, superimposes well with the protein molecule including the 6xHis-tag (Fig. 2).

### Electron microscopy

The nanoparticles were suspended in degassed water then dried onto glow discharged, carbon-coated copper TEM grids. The particles were imaged using the FEI Technai G2 Spirit electron microscope, equipped with camera. For HRTEM a FEI Titan microscope was used. All images were analysed using ImageJ^67^, by measuring the particle diameters of 100 particles. MmsF, shown as a comparison, was from 200 particles^24^. For shape analysis, we defined particles which had an aspect ratio ≥1:3 as needle, those <1:3 and with clear facets we defined as cuboidal, and particles with no clear definition (e.g. rounded) as irregular. Large (>300 nm) hexagonal plates, likely to be green rust, were discarded from the analysis as they could not be counted accurately.

### Powder X-ray diffraction and magnetometry

For X-ray analysis the nanoparticles were dispersed in water following the RTCP. The particles were dried down into a powder. Powder XRD patterns were collected using a standard X-ray diffractometer with Bragg–Brentano geometry (Bruker D8, CuK_α_ radiation). Scattering angle 2*θ* was scanned in a range of 20–80°. The reflections observed in the XRD patterns can be assigned to magnetite crystal structure.

For magnetometry, pre-weighed, dried samples of nanoparticles, were loaded into gelatine capsules. Data were recorded using an Oxford Instruments Maglab VSM operating at 55 Hz. The sample temperature was 290 K.

### Nanoparticle ELISA

100 µg of magnetite nanoparticles (NP) were blocked with 2× blocking buffer (Sigma Blocking solution) in PBS-T. 500 pmol of the coiled coil protein was added to each sample with binding for 1 h in a volume of 300 µl of 2× blocking buffer. The NP were washed with PBS-T before addition of rabbit anti-His6 antibody (Promokine) for 1 h. The NP were washed again, and Anti-Rabbit IgG alkaline phosphatase conjugate (Sigma Aldrich) was added. After 1 h the NP were washed with PBS-T three times and detected with Blue-Phos (KPL) reagent. After 15 min the particles were magnetically removed and the absorbance of the supernatant at 600 nm was measured. Blue coloured solution indicates binding, and yellow indicates no/little binding.

### Ferrous ion binding

3 µl of purified proteins (including ferritin as a positive control) were spotted onto nitrocellulose membrane at a concentration of 0.2 mg mL^−1^. After air drying, the membrane was blocked in 3% wt/vol bovine serum albumin in PBS-Tween buffer. After 1 h, 200 mM of ferrous chloride was added to the membrane in deoxygenated blocking solution and allowed to incubate for 3 h with gentle mixing. The membrane was then washed with PBS-Tween three times, and once with ultrapure water, to remove excess metal ions. The membrane was exposed to Clarity ECL detection reagent (Bio-Rad, UK) and visualised with a Chemi-Doc gel documentation system (Bio-Rad, UK). Bright spots indicate metal binding.

## Supplementary Information

Supplementary information

## Data Availability

The data/data sources that support the findings of this paper are contained/cited within the paper or Supplementary Information. Further raw data and biological materials are available from the corresponding author upon reasonable request.
