# Supplementary material for: Artificial coiled coil biomineralisation protein for the synthesis of magnetic nanoparticles
Source: Nat Commun. 2019 Jun 28;10:2873. doi: 10.1038/s41467-019-10578-2 (PMC6599041; doi:10.1038/s41467-019-10578-2)
Supplement: Supplementary file 1 — Supplementary information [file 41467_2019_10578_MOESM1_ESM.pdf]

Supplementary Information

Artificial coiled coil biomineralisation proteins for the synthesis of magnetic nanoparticles

Andrea E Rawlings,[1,2,†] Lori A Somner,[1†] Michaela Fitzpatrick-Milton,[1] Thomas P Roebuck,[3] Christopher Gwyn,[2] Panah Liravi,[3] Victoria E. Seville,[1] Thomas J Neal,[1] Oleksandr O. Mykhaylyk,[1] Stephen A Baldwin,[3] and Sarah S Staniland,[1,2,]

[1]Department of Chemistry, University of Sheffield, Brook Hill, Sheffield, S3 7HF
[2]School of Physics and Astronomy, University of Leeds, Leeds, LS2 9JT
[3]School of Biomedical Sciences, University of Leeds, Leeds, LS2 9JT

S.S.Staniland@sheffield.ac.uk

[†]These Authors contributed equally

.

Supplementary figures

Supplementary Figure 1

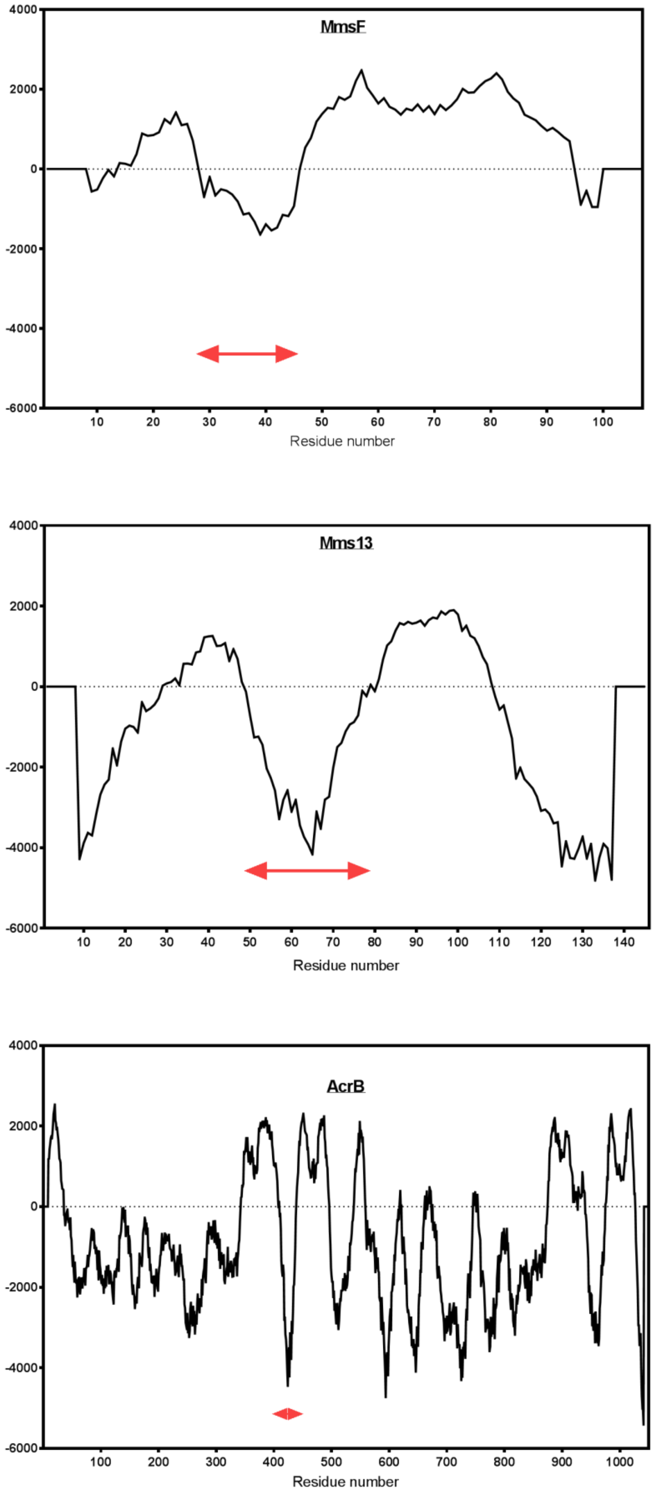

TMpred results for MmsF, Mms13 and AcrB, to show the predicted location of membrane spanning helices.[1] Y-axes are hydropathy index, and x-axes show residue position. Positive y-axis values indicate a particular residue will be membrane associated. Red lines indicate the loop transferred to the coiled coil scaffold.

Supplementary Figure 2

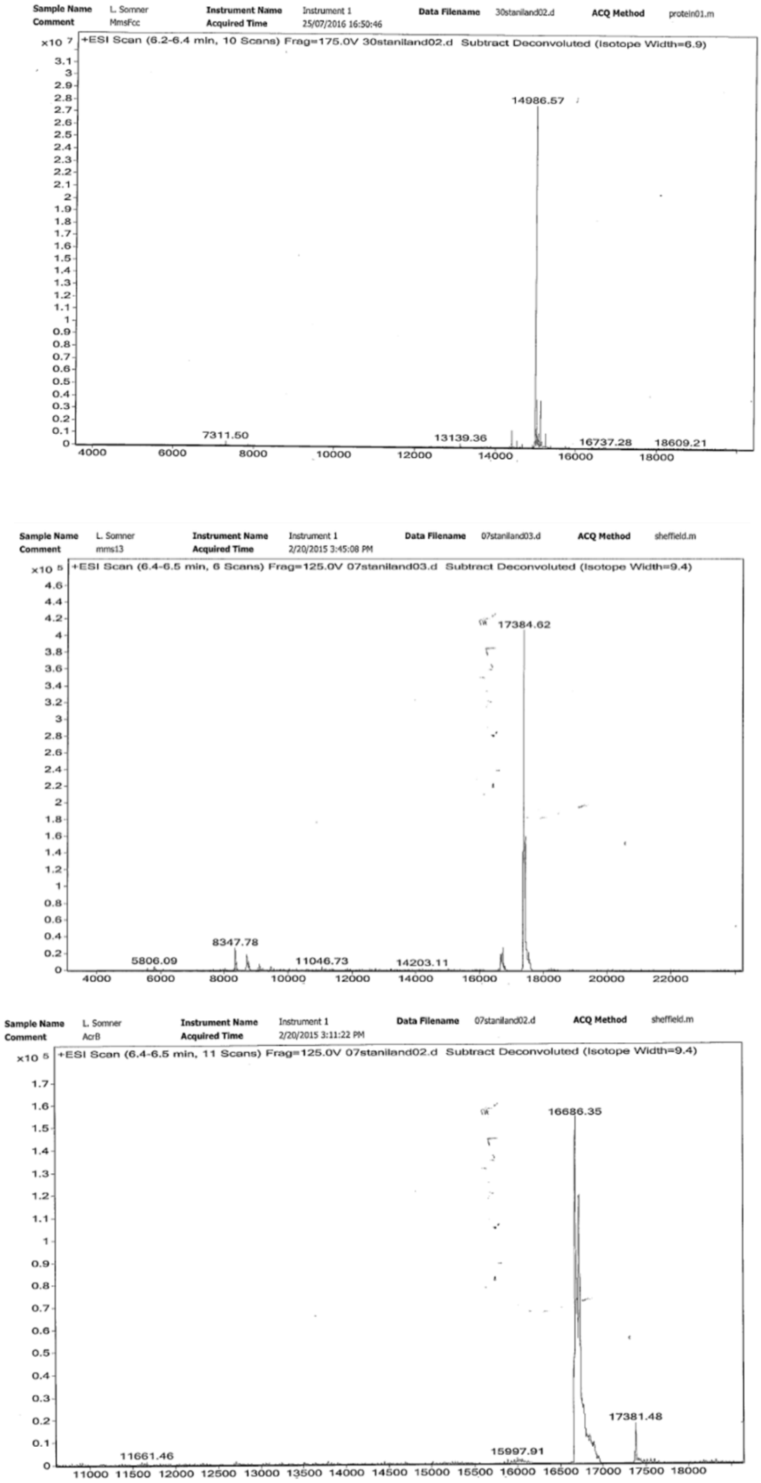

Raw mass spectrometry results of MmsFcc, Mms13cc, and AcrBcc respectively.

Supplementary Figure 3

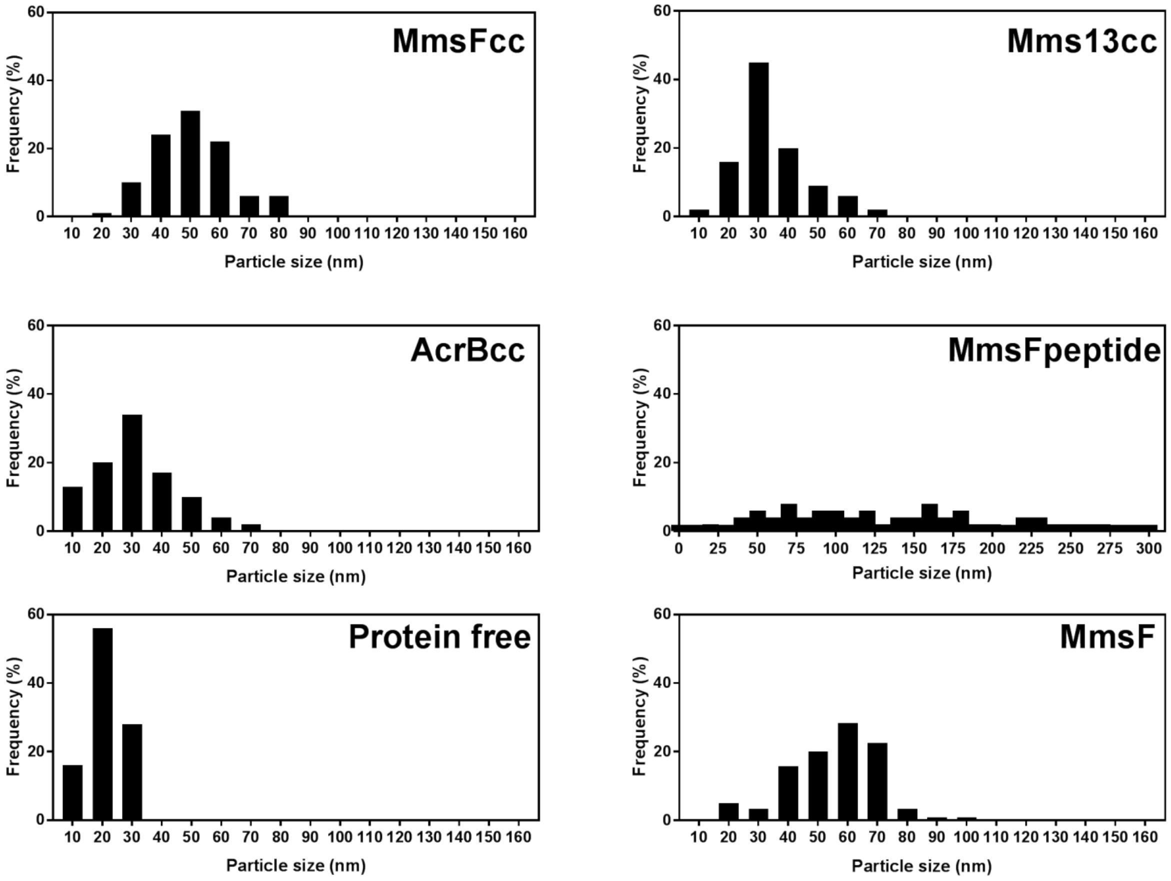

Grain size histogram of particles synthesised in the presence of 50 µg protein per 10 mL reaction. Protein free control particles are included for reference. X-axes are particle diameters (nm) and the frequency is the percentage of the particles measured. MmsFcc, Mms13cc, AcrBcc, MmsF peptide and protein free are from sizing of 100 particles each. MmsF at 100 µg per 10 ml reaction, 200 particles measured (see Rawlings *et al.*[2]).

Supplementary Figure 4

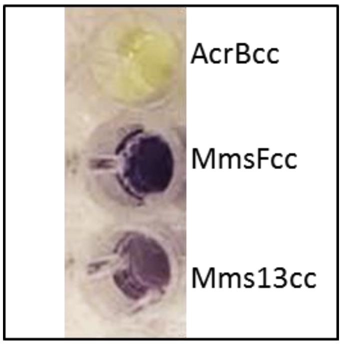

ELISA analysis of proteins added to pre-formed magnetite nanoparticles. Blue/purple colour indicates positive binding. Yellow represents limited/no binding.

Supplementary Figure 5

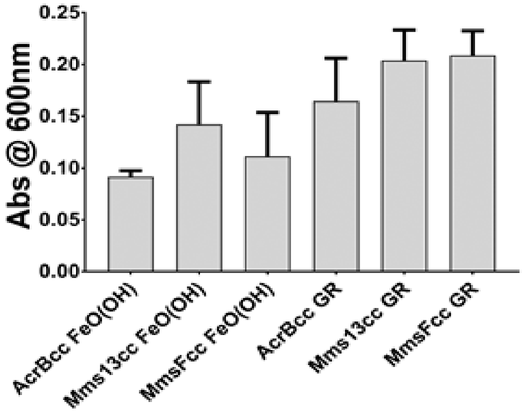

ELISA analysis of proteins added to ferric hydroxide (FeO(OH)) or green rust (GR). Samples analysed in triplicate. No statistically significant differences ($p=0.05$).

Supplementary Figure 6

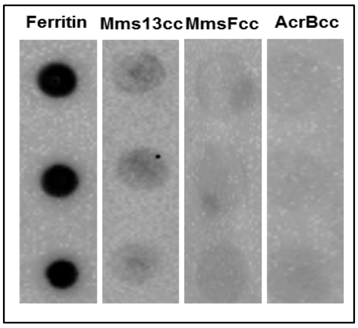

Blot based analysis of Fe(II) binding to protein samples. A dark spot indicates a positive binding result. Ferritin used as a positive Fe(II) binding protein. AcrBcc represents a negative control for the 6xHis tag. Samples analysed in triplicate.

<u>Supplementary Tables</u>

Supplementary Table 1

CC PROTEIN	MOLECULAR ION PEAK (m/z)	EXPECTED MASS (Da)
MmsFcc	14986	14986
Mms13cc	17384	17384
AcrBcc	16686	16686

The detected masses from mass spectrometry analysis, alongside the theoretical masses for the three proteins listed.

Supplementary Table 2

Comparison	Significant	P-value	Summary
MmsFcc v Protein free	Yes	<0.0001	****
Mms13 v Protein free	Yes	<0.0001	****
AcrBcc v Protein free	Yes	<0.0001	****
MmsFcc v Mms13cc	Yes	<0.0001	****
MmsFcc v AcrBcc	Yes	<0.0001	****
Mms13cc v AcrBcc	No	0.1084	ns
MmsFcc v MmsF peptide	Yes	<0.0001	****
Mms13cc v MmsF peptide	Yes	<0.0001	****
AcrBcccc v MmsF peptide	Yes	<0.0001	****
Protein free v MmsF peptide	Yes	<0.0001	****

Statistical analysis of particle sizing from TEM images. 1-way ANOVA performed using Graphpad Prism. ns indicates not significant.

Supplementary Notes

Supplementary Note 1

The sequence of the full length coiled coil protein (MmsFcc) is:
MGSHHHHHHHHGSTENLYFQGPSMKQLEKELKQLEKELQAIEKQLAQLQWKAQARKKKLA
QLKKKLQADRDDEFVYFHAKQGMKQLEKELKQLEKELQAIEKQLAQLQWKAQARKKKLA
QLKKKLQA

Supplementary References

1 Hoffman, K. & Stoffel, W. TMbase - A database of membrane spanning proteins segments. *Biol. Chem. Hoppe-Seyler,* **166** (1993).

2 Rawlings, A. E. *et al.* Self-assembled MmsF proteinosomes control magnetite nanoparticle formation in vitro. *Proc. Natl. Acad. Sci. U. S. A.* **111,** (2014).
